# Gender differences in $\dot{V}O_{2}$ and HR kinetics at the onset of moderate and heavy exercise intensity in adolescents

**DOI:** 10.14814/phy2.12970

**Published:** 2016-09-22

**Authors:** Nicola Lai, Alessandro Martis, Alfredo Belfiori, Fatima Tolentino‐Silva, Melita M. Nasca, James Strainic, Marco E. Cabrera

**Affiliations:** ^1^ Department of Biomedical EngineeringCase Western Reserve UniversityClevelandOhio; ^2^ Department of Pediatrics CardiologyCase Western Reserve UniversityClevelandOhio; ^3^ Center for Modeling Integrated Metabolic SystemsClevelandOhio; ^4^ Department of Electrical and Computer EngineeringOld Dominion UniversityNorfolkVirginia; ^5^ Biomedical Engineering Institute, Old Dominion UniversityNorfolkVirginia; ^6^ Rainbow Babies and Children's HospitalClevelandOhio

**Keywords:** adolescents, African American, exercise, heart rate, kinetics, modeling, pulmonary O_2_ uptake

## Abstract

The majority of the studies on $\dot{V}O_{2}$ kinetics in pediatric populations investigated gender differences in prepubertal children during submaximal intensity exercise, but studies are lacking in adolescents. The purpose of this study was to test the hypothesis that gender differences exist in the $\dot{V}O_{2}$ and heart rate (HR) kinetic responses to moderate (M) and heavy (H) intensity exercise in adolescents. Twenty‐one healthy African‐American adolescents (9 males, 15.8 ± 1.1 year; 12 females, 15.7 ± 1 year) performed constant work load exercise on a cycle ergometer at M and H. The $\dot{V}O_{2}$ kinetics of the male group was previously analyzed (Lai et al., Appl. Physiol. Nutr. Metab. 33:107–117, 2008b). For both genders, $\dot{V}O_{2}$ and HR kinetics were described with a single exponential at M and a double exponential at H. The fundamental time constant (*τ*_1_) of $\dot{V}O_{2}$ was significantly higher in female than male at M (45 ± 7 vs. 36 ± 11 sec, *P* < 0.01) and H (41 ± 8 vs. 29 ± 9 sec, *P* < 0.01), respectively. The functional gain (G_1_) was not statistically different between gender at M and statistically higher in females than males at H: 9.7 ± 1.2 versus 10.9 ± 1.3 mL min^−1^ W^−1^, respectively. The amplitude of the slow component was not significantly different between genders. The HR kinetics were significantly (*τ*_1_, *P* < 0.01) slower in females than males at M (61 ± 16 sec vs. 45 ± 20 sec, *P* < 0.01) and H (42 ± 10 sec vs. 30 ± 8 sec, *P* = 0.03). The G_1_ of HR was higher in females than males at M: 0.53 ± 0.11 versus 0.98 ± 0.2 bpm W^−1^ and H: 0.40 ± 0.11 versus 0.73 ± 0.23 bpm W^−1^, respectively. Gender differences in the $\dot{V}O_{2}$ and HR kinetics suggest that oxygen delivery and utilization kinetics of female adolescents differ from those in male adolescents.

## Introduction

The characterization of pulmonary oxygen uptake kinetics ($\dot{V}O_{2}$) response to exercise is used to evaluate cardiorespiratory and skeletal muscle function in exercise physiology (Whipp and Wasserman 1972; Linnarsson 1974; Whipp and Rossiter 2005). The $\dot{V}O_{2}$ response to constant work rate exercise reflects indirectly muscle oxygen (O_2_) consumption (Poole et al. 1991; Knight et al. 1992) and its parameter characteristics provide information on bioenergetic processes sustaining and limiting exercise performance which depends upon exercise intensity. Most of the studies (Rossiter 2011; Poole and Jones 2012) focused on the characterization of $\dot{V}O_{2}$ at different exercise intensities in adults (Cleuziou et al. 2004; Hughson 2005; Lai et al. 2012) and children (6–12 year olds) (Armon et al. 1991; Williams et al. 2001; Fawkner et al. 2002; Fawkner and Armstrong 2004b), whereas a few studies examined adolescent populations (Cooper et al. 1985; Lai et al. 2008b; Breese et al. 2010; Marwood et al. 2010; Salvadego et al. 2010). Several studies reported changes in $\dot{V}O_{2}$ with age (DeLorey et al. 2004; Fawkner and Armstrong 2004c; Armstrong and Barker 2009; Breese et al. 2010) and gender (Fawkner and Armstrong 2003, 2004b), but the physiological mechanisms regulating these changes during growth and maturation are yet to be fully uncovered (Fawkner and Armstrong 2003). Although some studies reported faster $\dot{V}O_{2}$ kinetics in children than adults (Macek and Vavra 1980; Fawkner et al. 2002), another study showed no differences in the kinetics (Hebestreit et al. 1998).

The underlying patterns of the $\dot{V}O_{2}$ kinetic responses to constant work rate exercise have been difficult to characterize in children, given the inherent low signal‐to‐noise ratio typical of their responses, especially at low exercise intensities (Armstrong and Barker 2009). However, the availability of breath‐by‐breath systems and mathematical modeling techniques have improved the characterization of the intensity‐dependent profiles of the $\dot{V}O_{2}$ responses to step transitions to work rates. Thus, $\dot{V}O_{2}$ responses were characterized in children (Armstrong and Barker 2009). In a prepubertal children study, the response to moderate‐intensity exercise was not different between boys and girls (Fawkner et al. 2002), whereas response to heavy‐intensity exercise was faster in boys than girls (Fawkner and Armstrong 2004b). The slow component in males was smaller than that in females although functional gain was not different between the two groups. Longitudinal studies on $\dot{V}O_{2}$ kinetics response to heavy‐intensity exercise in children showed that the primary time constant and slow component increase after a 2‐year period indicating an age‐dependent change of the muscle oxygen delivery and utilization (Fawkner and Armstrong 2004c; Breese et al. 2010) in pediatric population. Another study on gas exchange kinetics during incremental ramp exercise showed indirect evidences for oxygen delivery and utilization differences between healthy male and female pediatric groups (8–18 year) (Cooper et al. 2014). In obese adolescents, gender differences were also found at moderate‐intensity exercise, whereas $\dot{V}O_{2}$ kinetics in male was faster than female (Lee Franco et al. 2014).

The relationship between cardiovascular and respiratory responses was investigated in adult (Bearden and Moffatt 2001) and adolescent males (Barker et al. 2010a). This previous study on the influence of priming exercise on $\dot{V}O_{2}$ kinetics indicated that O_2_ delivery does not affect the time constant while it affects the overall $\dot{V}O_{2}$ kinetics. Another study in young and middle‐age adults suggested that $\dot{V}O_{2}$ kinetics response to heavy exercise is limited by slow heart rate (HR) and cardiac output kinetics (Hughson et al. 2000). In another NMR and NIRS study (Willcocks et al. 2010), the faster muscle oxygenation kinetics in children than adults in response to constant heavy exercise was attributed to a reduced ability of the children for oxygen delivery in comparison to adults. Gender‐related differences were also identified in the cardiovascular response to exercise in children (Armstrong and Welsman 2002). The steady‐state exercise HR during heavy‐intensity exercise was higher in girls than boys, whereas stroke volume was reduced in girls compared to boys. In females, HR value was reported to be higher than males at a given submaximal exercise intensity (O'Toole 1989). Other studies in children (Cooper et al. 1985) and adults (Lamarra 1982) showed that subjects with higher HR base line manifest slower HR kinetics. These findings would suggest a slower HR kinetics in females than male, although the effect of gender on HR kinetics at the onset of exercise was not determined.

These studies suggest, therefore, that gender differences exist in the ability to deliver and/or to utilize oxygen by the working muscle at different exercise intensity in children. Indeed, gender and maturation are important factors affecting $\dot{V}O_{2}$ kinetics (Armstrong and Welsman 2002), but only limited research (Fawkner and Armstrong 2004b,c; Armstrong and Barker 2009; Willcocks et al. 2010) focused on gender differences in the cardiorespiratory response to a step change in work rate at different exercise intensities in adolescents. Therefore, the purpose of this study was to test the hypothesis that there are gender‐related differences in the $\dot{V}O_{2}$ and HR kinetics responses to moderate‐ and heavy‐intensity exercise and whether there is an association between $\dot{V}O_{2}$ and HR kinetics in adolescents.

## Materials and methods

### Subjects

Nine male and twelve female healthy African‐American adolescents were included in the study; all the subjects were nonsmokers, taking no medications, and were not involved in competitive athletics and regular exercise training at the time of the study. The analysis of the $\dot{V}O_{2}$ kinetics of nine male African‐American adolescents was previously determined (Lai et al. 2008b). All procedures were approved by the University Hospitals of Cleveland Institutional Review Board and written informed consents were obtained from both the subjects and their parents.

### Measurements

The anthropometric measurements were obtained just 1 day before the maximal exercise test. Stature was measured with a standard, calibrated stadiometer (Seca, Vogel and Halke, Hamburg, Germany) and body mass with a balance beam scale (Seca, Vogel and Halke). Stage of maturation was estimated by self‐assessment method proposed by Morris and Udry (Morris and Udry 1980) and used to confirm that all subjects were postpubertal (Stage 4–5). All subject characteristics are reported on Table 1.

**Table 1:** Subjects characteristics by gender

	Male	Female
*n*	9	12
Age (year)	15.8 ± 1.1	15.7 ± 1
Height (m)	1.76 ± 0.05^a^	1.62 ± 0.06
Weight (kg)	69.5 ± 10.5^a^	55.2 ± 7.5
BMI (kg m^−2^)	22.4 ± 2.6	21 ± 2.6
$\dot{V}O_{2\mathrm{peak}}$ (L min^−1^)	3.06 ± 0.44^a^	1.8 ± 0.3
$\dot{V}O_{2\mathrm{peak}}$ (mL kg^−1^ min^−1^)	44.4 ± 6.2^a^	32.5 ± 3
HR_peak_ (bpm^1^)	190 ± 8	191 ± 6
WR at $\dot{V}O_{2\mathrm{peak}}$ (W)	222 ± 29^a^	142 ± 18
LT_GE_ (L min^−1^)	1.56 ± 0.28^a^	0.89 ± 0.16
LT_GE_ (mL kg^−1^ min^−1^)	22.3 ± 3.6^a^	16.2 ± 2.4
WR at LT_GE_ (W)	115 ± 23^a^	73 ± 14

a *P *<* *0.05 significantly different from female.

The exercise tests were performed on an electronically braked cycle ergometer (Ergometrics 800, Sensor‐Medics; Yorba Linda, CA) at approximately the same time of day. The subjects were reported to the laboratory on four occasions within a 2‐week period. They were instructed to refrain from eating and exercising in the 2 hours prior to their scheduled exercise tests. The experimental procedures were the same of those used to study the male adolescents. In the first experiment, the subjects performed a 20 W min^−1^ incremental ramp test until they reached exhaustion for determination of peak $\dot{V}O_{2}$ ($\dot{V}O_{2\mathrm{peak}}$) and gas exchange threshold (LT_GE_) via respiratory measurements (Beaver et al. 1986). The delta parameter (Δ = $\dot{V}O_{2\mathrm{peak}}$−LT_GE_) and LT_GE_ were used to determine the individual work rates for each of the exercise intensity domains investigated. On the subsequent visits, the subjects performed a series of six square‐wave exercise tests; two per day, at selected work rates, which corresponded to: (a) 90% LT_GE_ (moderate, M) and (b) LT_GE_ + 25% of Δ (heavy, H). Each subject exercised three times at the moderate and heavy intensities. The tests were preceded by a 3‐min baseline period and a 3‐min warm‐up period at 20 W. At the end of the test, the work rate was abruptly reduced to 20 W for a 10 min (active recovery period) and followed by an additional 5 min (passive recovery) while the subjects remained seated quietly on the cycle. The pedaling rate was kept constant at 60 rpm for all exercise intensity tests. During moderate exercise, subjects exercised for 5 min at the predetermined work rate. During heavy exercise, subjects were asked to pedal until they had achieved a steady state which was defined as 2 min of a <5% change in $\dot{V}O_{2}$ and a <3% change in HR and respiratory exchange ratio.

Instructions to begin and end testing were given by voice without warning. ‘‘Steady‐state’’ values were calculated by averaging data recorded over the last 30 sec of exercise. All square‐wave tests performed were assigned in a randomized sequence to avoid ordering effects. A break of 60–90 min was enforced between exercise bouts conducted on the same day.

A facemask (8940 Series, Hans Rudolph, Inc.; Kansas, MO) was carefully fitted and sealed with a gel (Hans Rudolph, Inc.) before the exercise and data collection to obviate any gas leaks. The subjects were given several minutes to familiarize themselves with the breathing apparatus in order to minimize unusual breathing patterns. To measure gas exchange, subjects breathed through a mass flow sensor (hot‐wire anemometer) connected to a metabolic cart system (VMax 29, SensorMedics, Yorba Linda, CA). Before each exercise test, the volume sensor was calibrated using a 3‐L syringe while the O_2_ and CO_2_ analyzers were calibrated as previously reported (Lai et al. 2008b). Before, during, and after exercise and recovery, ventilatory, and metabolic variables [ventilation (${\dot{V}}_{E}$), pulmonary oxygen uptake ($\dot{V}O_{2}$), and carbon dioxide release ($\dot{V}{\text{CO}}_{2}$)] were continuously monitored. These measurements permitted the determination of the ventilatory equivalents for O_2_ (*V*_E_/$\dot{V}O_{2}$) and CO_2_ (${\dot{V}}_{E}$/$\dot{V}{\text{CO}}_{2}$) as well as the respiratory exchange ratio ($\dot{V}{\text{CO}}_{2}$/$\dot{V}O_{2}$). A three‐lead electrocardiogram (SensorMedics) was continuously displayed and used to record HR during the test. Systemic systolic/diastolic blood pressure was measured every 3 min during the maximal exercise test with an automated cuff system (Tango, SunTech Inc., Morrisville, NC).

### Data processing, modeling, and dynamic analysis

The $\dot{V}O_{2}$and HR kinetics data from individual repetitions of moderate and heavy exercise intensities were processed before the estimates of the parameter values. First, the data values greater than 2 SD from their local mean were omitted from those used for parameter estimation. Second, the responses for each trial were linearly interpolated to obtain a value at each second. Corresponding values on a second‐by‐second basis were then ensemble averaged to produce a mean dynamic response. Then, averaged values every 5 sec were calculated and utilized for kinetic analysis. The data obtained during the first 20 sec were excluded from the analysis (Ozyener et al. 2001; Breese et al. 2010) for both $\dot{V}O_{2}$ and HR kinetics. The cardiorespiratory responses to square‐wave change in work rate were characterized with the exponential models listed below using the nonlinear curve fitting function (‘‘lsqcurvefit’’) available in Matlab (The Mathworks, Natick, MA) customized and applied to the mean responses. The following models were used:

Model 1:

$$\Delta Y\left(t\right)=Y\left(t\right)-Y_{\mathrm{BL}}=A_{1}\left(1-e^{\left(-\frac{t-\delta_{1}}{\tau_{1}}\right)}\right)H\left(t-\delta_{1}\right)$$

Model 2:

$$\Delta Y\left(t\right)=Y\left(t\right)-Y_{\mathrm{BL}}=A_{1}\left(1-e^{\left(-\frac{t-\delta_{1}}{\tau_{1}}\right)}\right)H\left(t-\delta_{1}\right)+A_{2}\left(1-e^{\left(-\frac{t-\delta_{2}}{\tau_{2}}\right)}\right)H\left(t-\delta_{2}\right)$$

where *Y* = $\dot{V}O_{2}$ and HR, $Y_{\mathrm{BL}}$ represents the steady‐state values at baseline (i.e., warm up); *A*_1_ and *A*_2_ are the amplitudes of the exponential terms; *τ*_1_ and *τ*_2_ are the time constants; and *δ*_1_ and *δ*_2_ are the time delays. The Heaviside step function (Abramowitz and Stegun 1972) *H*(*t*−*δ*_1_) and H(*t*−*δ*_2_) are used to constrain the exponential terms to their corresponding time domains. Subscripts ‘‘1’’ and ‘‘2’’ denote the fast (or fundamental) and slow components of $\dot{V}O_{2}$ and HR dynamic response, respectively. From the fundamental and slow‐phase amplitudes (*A*’_1_, *A*’_2_) and the change in work rate from baseline (ΔWR), the functional gains of the primary response ($G_{1}=A_{1}^{\prime }/\delta \text{WR}$) and the end‐exercise response ($G_{\mathrm{TOT}}=\left[A_{1}^{\prime }+A_{2}^{\prime }\right]/\delta \text{WR}$) were calculated. Both models (mono and double exponential) were used to characterize each dataset obtained at moderate and heavy exercise intensities.

The interval of time to reach half of the amplitude (*t*_1/2_, half time) of the $\dot{V}O_{2}$ and HR kinetics was determined to assess the relationship between $\dot{V}O_{2}$ and HR responses to moderate and heavy exercise intensities in both adolescent groups.

### Statistical analyses

All data are expressed as means ± SD. Comparison of estimated kinetic parameters within a group were performed using a mixed model analysis of variance accounting for repeated measures on each subject. Post hoc analysis with a t‐test and least significant differences was used to discern differences in the parameters among intensity domains. An F‐test was performed to evaluate whether the data were fit significantly better by model 2 than model 1. The square of the correlation coefficient was used to evaluate the goodness of fit for each model. Pearson's correlation was used to assess a linear association between selected variables. For all tests, a *P* value 0.05 was considered statistically significant. Statistical analyses were performed using Sigma Stat software (Sigma stat 2.03, SPSS, San Jose, CA).

## Results

The female group was selected to match the age and body mass index of the male group previously investigated (Lai et al. 2008b). Both adolescent groups had similar normal body weight and physical activity. The cardiorespiratory responses to the ramp exercise test are reported in Table 1. The peak of $\dot{V}O_{2}$ and peak of $\dot{V}O_{2}$ normalized to the body weight of the female group were 59 and 73% of that of the male group, respectively. The WR in female was 64% of that of the male group, whereas the peak of HR (191 ± 6 bpm) was similar in both adolescent groups. $\dot{V}O_{2}$ and WR at the LT_GE_ were significantly higher (*P* < 0.05) in males. In females as compared to males, the $\dot{V}O_{2}$ at the LT_GE_ was 0.89 ± 0.16 versus 1.56 ± 0.28 L min^−1^ and occurred at work rates of 73 ± 14 and 115 ± 23 W, respectively.

**Figure 1:**
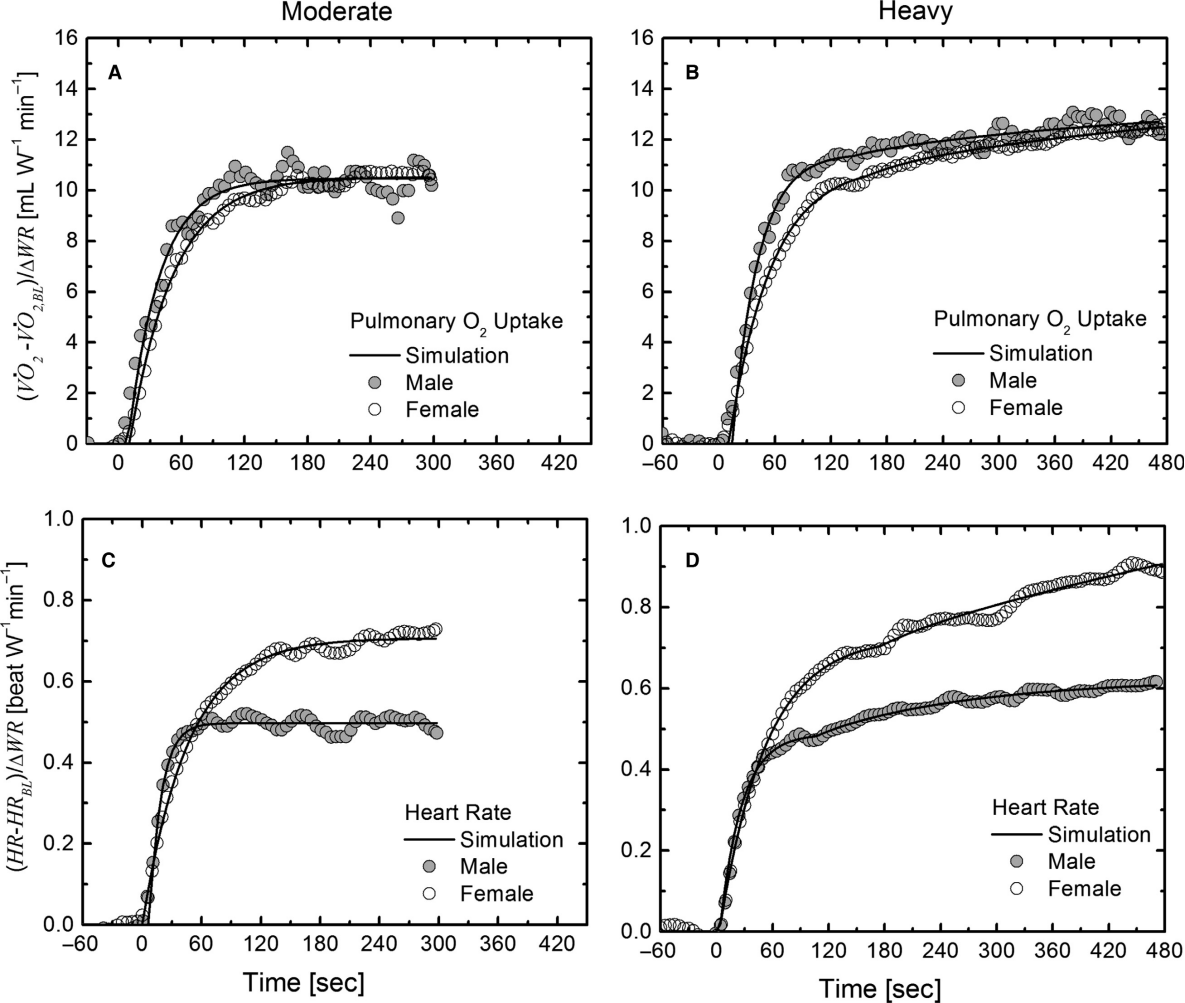
Comparison of model simulations (line) and experimental data of mean $\dot{V}O_{2}$ (A, B) and heart rate (C, D) dynamic response to constant work rate exercise of moderate and heavy intensity in a representative female (open circle symbols) and male (closed circle symbols) adolescent. The graphs of the mean responses are showed with 5 sec intervals of time and ensemble averages of interpolated and time‐aligned breath‐by‐breath data from individual transitions from warm up at 20 W. The subscript BL indicates the steady‐state value at the warm up.

The $\dot{V}O_{2}$ and HR time profiles followed the general patterns reported in Figure 1. The steady‐state values for warm up, moderate (M), and heavy (H) exercise intensity were reported in Table 2, whereas the parameters of the $\dot{V}O_{2}$ and HR kinetics were estimated with nonlinear curve fitting of exponential models (i.e., 1 and 2) and reported in Table 2. It should be noted that only the $\dot{V}O_{2}$ kinetics of the male group were estimated and analyzed in another previous study (Lai et al. 2008b).

**Table 2:** Effect of exercise intensity on kinetic parameters^d^ of the $\dot{V}O_{2}$ and HR responses to square‐wave exercise of moderate (M) and heavy (H) intensity by gender

	Male		Female	
	M	H	M	H
Exercise duration (min)	5.4 ± 0.04	7.8 ± 0.6	5.4 ± 0.04	7.6 ± 0.6
Work rate (W)	102 ± 20^a^^,^ b	140 ± 23^a^	63 ± 13^b^	87 ± 15
Pulmonary oxygen uptake, $\dot{V}O_{2}$				
$\dot{V}O_{2,\mathrm{BL}}$	0.53 ± 0.08^a^	0.53 ± 0.06^a^	0.41 ± 0.04	0.40 ± 0.05
$\dot{V}O_{2,E}$	1.45 ± 0.3^a^^,^ b	2.0 ± 0.3^a^	0.93 ± 0.16^b^	1.3 ± 0.2
*A*_1_ (L min^−1^)	0.93 ± 0.3^a^^,^ b	1.2 ± 0.3^a^	0.51 ± 0.15^b^	0.75 ± 0.14
* A*_2_ (L min^−1^)		0.22 ± 0.1		0.13 ± 0.06
*δ*_1_ (s)	15 ± 2^a^	17 ± 2^a^	9.2 ± 3.9^b^	12.4 ± 2.7
*δ*_2_ (s)		115 ± 32		151 ± 47
*τ*_1_ (s)	36 ± 11^a^^,^ b [27÷44]	29 ± 9^a^ [23÷35]	45 ± 7[41÷49]	41 ± 8[36÷46]
*τ*_2_ (s)		197 ± 92		283 ± 106
G_1_ (mL min^−1 ^W^−1^)	10.9 ± 0.9^c^	9.7 ± 1.2^a^	11.3 ± 1.2^c^	10.9 ± 1.3
G_TOT_ (mL min^−1 ^W^−1^)		11.6 ± 0.9^a^		12.8 ± 1.0
Heart rate, HR				
HR_BL_	93 ± 7^a^	91 ± 9^a^	104 ± 7	106 ± 6
HR_E_	136 ± 10^a^^,^ b	162 ± 10^a^	148 ± 10^b^	178 ± 9
*A*_1_ (bpm)	42 ± 10	47 ± 13	43 ± 9^b^	53 ± 11
*A*_2_ (bpm)		21 ± 9		18 ± 9
*δ*_1_ (s)	0.9 ± 1.9^b^	4 ± 3.8	0.3 ± 0.9	1.8 ± 2.5
*δ*_2_ (s)		94 ± 37^a^		135 ± 46
*τ*_1_ (s)	45 ± 20^a^^,^ b [30÷61]	30 ± 8^a^[23÷36]	61 ± 16^b^ [51÷71]	42 ± 10[36÷48]
*τ*_2_ (s)		245 ± 115		223 ± 75
G_1_ (bpm W^−1^)	0.53 ± 0.11^a^^,^ b	0.40 ± 0.11^a^	0.98 ± 0.2^b^	0.79 ± 0.26
G_TOT_ (bpm W^−1^)		0.58 ± 0.13^a^		1.04 ± 0.22

The confidence interval is enclosed between square brackets.

a *P* < 0.05 significant gender difference at the same exercise intensity.

b *P* < 0.05 significant exercise intensity within group.

c *P* < 0.05 significant different from G_TOT_ within group.

d The subscript BL and E indicate the steady‐state value at the warm up and end of exercise, respectively.

### $\dot{V}O_{2}$ *kinetics*

In both adolescent groups, the kinetics response at M was well fitted by a monoexponential model (i.e., Model 1), whereas that at H by a double‐exponential model (i.e., Model 2) according to the F‐test. The mean $\dot{V}O_{2}$ value at the end of the transition period ($\dot{V}O_{2,E}$) increased with increasing exercise intensity in both groups (Table 2). Between groups, however, males had a $\dot{V}O_{2,E}$ higher than females at M and H. The amplitude of the fundamental phase (*A*_1_) increased with exercise intensity in both groups (Table 2). Between the groups, *A*_1_ was significantly greater in males than in females at each intensity exercise. The time delay (*δ*_1_) differed significantly among the two exercise intensity domains only for the females but not for the males, moreover, it differed between groups at any intensity. The fundamental time constant (*τ*_1_) was independent of exercise intensity only for the females. In both groups, a $\dot{V}O_{2}$ slow component became evident after approximately 2–3 min (*δ*_2_) of the phase II of exercise responses at H. The mean value of the Phase III time constant of the response (*τ*_2_) was not different between the two gender (183 ± 97 for the males vs. 288 ± 103 sec, *P* < 0.56). The exhaustion time of the heavy exercise test for females (7.6 ± 0.6 min) was similar to that observed in males (7.8 ± 0.6 min). The magnitude of the functional gain of the fundamental phase (G_1_) in males was similar to that measured in females at moderate intensity while was lower than females at heavy exercise intensity. Moreover, there was no gender difference in G_TOT_ at M, whereas it was found lower in males than females at H.

There was no correlation between the *τ*_1_ of $\dot{V}O_{2}$ and $\dot{V}O_{2,\mathrm{peak}}$ for male (*r *=* *−0.085, *P *=* *0.8) and female (*r *=* *−0.5, *P *=* *0.1) adolescents.

### HR kinetics

The HR kinetics as for the $\dot{V}O_{2}$ kinetics were characterized by the single‐and double‐exponential model for M and H, respectively. The HR mean value at the end of the exercise (HR_E_) increased by 20% with exercise intensity in both groups (Table 2). The females had a HR_E_ value 10% significantly higher than males at both exercise intensities. The amplitude *A*_1_ increased with exercise intensity but between groups, it was similar in males and females at each corresponding intensity. The *τ*_1_ in males was 35–40% smaller than that estimated in females at M and H. Within each group, the *τ*_1_ decreased with exercise intensity by approximately 32%. A HR slow component became evident after approximately 94 and 135 sec (*δ*_2_) of the Phase II of exercise response at H for males and females, respectively. The mean value of the *τ*_2_ was similar for both gender (245 ± 115 sec, males vs. 223 ± 75 sec, females). In males, the G_1_ and G_TOT_ were approximately 50% lower than that determined in females at M and H. The G_TOT_ was independent from the exercise intensity for both gender groups.

To assess the relationship between $\dot{V}O_{2}$ and HR kinetics, a linear association between *τ*_1_ of $\dot{V}O_{2}$ and HR kinetics was investigated in both groups of adolescents (Table 3). In males, the correlation between *τ*_1_ of $\dot{V}O_{2}$ and HR kinetics was significant at M (*r *=* *0.8, *P *<* *0.01) and was not significant at H (*r *=* *−0.32, *P *=* *0.4), whereas in females the correlation between *τ*_1_ of $\dot{V}O_{2}$ and HR was not significant at both exercise intensities (M: *r *=* *0.2, *P *=* *0.5; H: *r *=* *−0.3, *P *=* *0.3). The correlation between t_1/2_ of $\dot{V}O_{2}$ and HR kinetics was also investigated in both adolescent groups. In males, a significant correlation was observed at M (*r *=* *0.76, *P *=* *0.02) and H (*r *=* *0.92, *P *<* *0.01), whereas in females the correlation was not statistically significant at both exercise intensities (M: *r *=* *0.03, *P *=* *0.9; H: *r *=* *−0.13, *P *=* *0.7).

**Table 3:** Relationship between $\dot{V}O_{2}$ and HR responses to square‐wave exercise

		$\tau_{1,\dot{V}O_{2}}\;\text{vs.}\;\tau_{1,\mathrm{HR}}$	$t_{1/2,\dot{V}O_{2}}\;\text{vs.}\;t_{1/2,\mathrm{HR}}$
Group (21)	M	*r *=* *0.6, *P *<* *0.002	*r *=* *0.3, *P *=* *0.2
	H	*r *=* *0.2, *P *=* *0.5	*r *=* *0.4, *P *=* *0.08
Male (9)	M	*r *=* *0.8, *P *<* *0.01	*r *=* *0.76, *P *<* *0.03
	H	*r *=* *−0.32, *P *=* *0.4	*r *=* *0.92, *P *<* *0.01
Female (12)	M	*r *=* *0.2, *P *=* *0.5	*r *=* *0.03, *P *=* *0.9
	H	*r *=* *−0.3, *P* = 0.3	*r *=* *−0.13, *P *=* *0.7

## Discussion

The majority of the studies of $\dot{V}O_{2}$ kinetics in pediatric populations have investigated age and gender differences in prepubertal children during submaximal high‐intensity exercise, but studies are lacking in adolescents. In this study, $\dot{V}O_{2}$ and HR responses to constant work rate exercise of moderate and heavy intensities were characterized in females and compared to those of African‐American male adolescents. The main findings of this study showed gender differences in the $\dot{V}O_{2}$ and HR kinetic response to moderate and heavy exercise intensity in adolescents: (1) the primary time constant of $\dot{V}O_{2}$ and HR kinetics in females is lower than that of males; (2) the primary gain of $\dot{V}O_{2}$ kinetics is independent of exercise intensity for both groups, whereas it is greater in females than males only at heavy exercise intensity while the primary gain of HR kinetics is higher in female than male; (3) there was no gender difference in the slow component of $\dot{V}O_{2}$ kinetics; and (4) $\dot{V}O_{2}$ and HR kinetics were significantly correlated only for the male adolescent group.

There are no differences between boys and girls on the number of exponentials required to characterize $\dot{V}O_{2}$ kinetics response to moderate and heavy exercise. This result confirms that not only boys (Lai et al. 2008b) but also girls require a single‐exponential model for M and a double‐exponential model for H, respectively. This finding is in agreement with several studies conducted in children and adults (Linnarsson 1974; Whipp et al. 1982; Cooper et al. 1985; Ozyener et al. 2001; Fawkner and Armstrong 2004a; Lai et al. 2012). Nevertheless, in our study, *τ*_1_ of adolescents appears closer to the adult rather than prepubertal children's response to both M and H. This could be related to the maturation level that was reported to affect $\dot{V}O_{2}$ kinetics (Babcock et al. 1994; Fawkner and Armstrong 2004c; Armstrong and Barker 2009). In particular, *τ*_1_ was reported smaller in prepubertal children than adults (Fawkner et al. 2002). In females, *τ*_1_ was greater than males at M in accord with the results found in healthy (Cooper et al. 1985) and obese (Lee Franco et al. 2014) adolescents. At H, the *τ*_1_ was also greater in female than male in agreement with a previous study in children (Fawkner and Armstrong 2004b) and in contrast to another study from the same research group that did not observe gender differences (Fawkner and Armstrong 2004c). It was reported that fitness level can affect both $\dot{V}O_{2}$ kinetics parameters (G_1_ and *τ*_1_) (Barstow et al. 1996; Pringle et al. 2003; Boone et al. 2008) regardless of potential gender differences. In both females and males, the relationship between *τ*_1_ of $\dot{V}O_{2}$ and $\dot{V}O_{2\mathrm{peak}}$ was not statistically different indicating that the aerobic capacity of the male group is not responsible for the difference in the *τ*_1_ of $\dot{V}O_{2}$ between male and female groups. Thus, it is unlikely that the fitness level of the male group is related to the gender differences between the two adolescent groups.

At the onset of exercise, the systemic oxygen delivery adjustments to exercise also rely on HR changes which are greater in females than males (Table 2). Both amplitude and time constant parameters of the HR kinetics are important for the oxygen delivery. The HR kinetics difference between males and females could be responsible for gender differences in oxygen delivery and utilization kinetics at both exercise intensities. The slower $\dot{V}O_{2}$ kinetics in females than males could be in part related to the slower HR kinetics in females than males as *τ*_1_ of $\dot{V}O_{2}$ and HR kinetics in females were greater than those observed in male adolescents (Table 2). To further support this hypothesis, a correlation analysis was performed to determine the existence of a relationship between *τ*_1_ of $\dot{V}O_{2}$ and that of HR kinetics. In males, a significant relationship was found between these two parameters for M, whereas it was not significant for H. The t_1/2_ of $\dot{V}O_{2}$ and HR kinetics were also determined to have information on the overall response to heavy exercise, as *τ*_1_ parameter quantifies only the primary phase of the kinetics. Thus, a strong relationship between t_1/2_ of $\dot{V}O_{2}$ and HR kinetics was observed for both exercise intensities in male adolescent (Table 3). The correlation analysis on t_1/2_ and *τ*_1_ indicates that the overall $\dot{V}O_{2}$ kinetics may be affected by the cardiovascular system at both exercise intensities, but the phase II $\dot{V}O_{2}$ kinetics is independent from HR kinetics at H. The results observed at H appeared consistent with the evidences reported in two studies on the effect of priming exercise on $\dot{V}O_{2}$ kinetics at very heavy exercise in boys (Barker et al. 2010a, 2014). In these studies, the priming exercise intervention appeared to increase the amplitude and speed of the overall $\dot{V}O_{2}$ kinetics while it did not affect the phase II $\dot{V}O_{2}$ tau.

In contrast to the finding for males, there was no evidence for a significant relationship between $\dot{V}O_{2}$ and HR kinetics at both exercise intensities in females. We do not have a clear explanation for this observation. It should be noted that a significant relationship between *τ*_1_ of $\dot{V}O_{2}$ and HR kinetics was found at M when both groups were considered in the correlation analysis. Therefore, it cannot be excluded that the absence of association between $\dot{V}O_{2}$ and HR kinetics at M is related to the sample size. The only study in boys and girls that investigated the relationship between $\dot{V}O_{2}$ and HR kinetics (Cooper et al. 1985) reported no gender difference, although the sample size was quite small. In the same work, a linear relationship between HR base line and half time of the HR kinetics was also observed. Consistent with this observation, in our study, the HR base line was higher in females than that observed in males while the HR kinetics were slower in females than males at M and H (Table 2).

Beside the *τ*_1_ difference between males and females, the HR change relative to the workload (ΔHR/ΔWR) of males was 50% lower than females for both moderate and heavy exercise intensities. As the total gain Δ$\dot{V}O_{2}$/ΔWR in males was only 5–12% significantly lower than females, the corresponding Δ$\dot{V}O_{2}$/ΔHR in males was 78 and 61% higher than those in females at moderate and heavy exercise intensities, respectively. The higher total gain of HR of female in comparison to that found in male at both exercise intensity suggest that gender differences exist in the cardiovascular regulation during exercise. This result suggests a difference in stroke volume and/or oxygen extraction between male and female. Previous studies showed that the higher HR during exercise in females than males is related to regulatory mechanisms of the cardiovascular system to compensate the lower stroke volume changes in girls than boys (Armstrong and Welsman 2002) and women than men, (Wheatley et al. 2014). Nevertheless, HR kinetics provided only indirect information on systemic oxygen delivery, thus, specific studies should focus on muscle blood flow kinetics during exercise.

Although gender difference observed on HR kinetics is substantial at both exercise intensity, it cannot be excluded that their effect on $\dot{V}O_{2}$ kinetics at moderate was different than that at heavy exercise intensity. For M condition, several studies attributed the $\dot{V}O_{2}$ kinetic regulation to the bioenergetic processes at cellular level rather than central or peripheral oxygen delivery limitations which appear to be relevant only at H or above (Grassi 2000; Hughson 2005). A previous study on the effect of exercise intensity on $\dot{V}O_{2}$ kinetics in adults reported as indirect evidence of oxygen delivery limitation at H, a slower HR kinetics at H than M (McNarry et al. 2012). In the same study, *τ*_1_ and G_1_ of $\dot{V}O_{2}$ were found dependent on exercise intensity. In contrast, our results showed a G_1_ of $\dot{V}O_{2}$ independent of exercise intensity in females similarly to the previous finding on male adolescents (Lai et al. 2008b) indicating that oxygen delivery and utilization in adolescents differ from adults. Another work on the effect of training status on $\dot{V}O_{2}$ kinetics supports our results, suggesting that this kinetics are limited by oxygen delivery in adolescents (Marwood et al. 2010). The linear relationship between amplitude and work rate at intensities below and above the ventilatory threshold found in female adolescents confirmed previous results reported from our group for male adolescents (Lai et al. 2008b). Therefore, these results are consistent with study in adults (Barstow and Jones 1991), although there is no consensus whether G_1_ parameters are linearly related to the work rate from rest to $\dot{V}O_{2\mathrm{peak}}$ (Whipp and Wasserman 1972; Korzeniewski and Zoladz 2004; Wilkerson et al. 2004; Hughson 2005).

Several studies attributed the differences observed in G_1_ and *τ*_1_ between M and H to specific recruitment of type I and II fibers during muscle contraction in adults (Barstow et al. 1996; Pringle et al. 2003). In an adolescents study, although fiber content was not quantified, the effect of pedal rate on $\dot{V}O_{2}$ kinetics (Breese et al. 2011) suggested a muscle fiber recruitment similar to that observed in adults (Barstow et al. 1996) at the onset of exercise. In adults, the fraction of type I fibers was negatively correlated with *τ*_1_ (Pringle et al. 2003), whereas it was positively correlated with G_1_ (Pringle et al. 2003; Barker et al. 2010a). Therefore, the slower $\dot{V}O_{2}$ kinetics of female in comparison to male adolescents are consistent with a lower percentage of type I muscle fibers in girls than boys as previous reported on a study in adolescents (Glenmark et al. 1992). In contrast with the assumption of a lower percentage of type I muscle fibers in girls than boys, G_1_ was similar in females and males at M while it was even higher in females than males at H. The relationship between G_1_ and type I muscle fiber reported in previous studies (Pringle et al. 2003; Barker et al. 2010a) should be interpreted with caution as it appears in conflict with other studies that reported a strong correlation between cycling efficiency and type I muscle fiber (Coyle et al. 1992) and a higher muscle phosphate cost in type II than type I muscle fiber (Crow and Kushmerick 1982). According to this evidence, G_1_ is expected to increase with higher amounts of type II muscle fiber as G_1_ is related to the phosphate cost and P/O_2_ ratio (Rossiter 2011). NMR studies in adolescent (Barker et al. 2010b) indicated that gender differences emerge during high‐intensity exercise and could be related to muscle fiber recruitment based on the greater anaerobic component observed in girls compared to boys. Another NMR study in adolescents (Willcocks et al. 2010) confirmed that girls have a lower muscular efficiency than boys based on the higher phosphate cost observed in girls than boys. Therefore, this gender differences should not be related to a higher oxygen cost of phosphate production but rather to a higher phosphate cost (Rossiter et al. 2002) in female than male at exercise intensity above the ventilatory threshold.

The slow component observed in female adolescents was consistent with that observed in male adolescent (Lai et al. 2008b) and adults (Gaesser and Poole 1996). The similar amplitude of the slow component between the two adolescent groups suggests no gender differences in the recruitment of the fiber types at H. Therefore, gender differences in G_1_ and *τ*_1_ observed cannot be explained only by muscle fiber distribution but rather by a combination of factors related to gender differences in biochemical properties, oxygen delivery, and pattern of the recruitment of muscle fiber at moderate and heavy exercise intensity.

The results of this study should also be interpreted in light of several considerations reported in the following paragraphs. The relationship between $\dot{V}O_{2}$ and HR kinetics found in male provides only indirect evidence for impairment of systemic oxygen delivery. Measurement of cardiac output kinetics provides quantitative information to investigate the relationship between oxygen delivery and utilization at the onset of exercise. Nevertheless, cardiac output kinetics are quite close to those observed for HR as the stroke volume varies during the first 10–30 sec of the onset of a constant workload exercise (Miyamoto et al. 1982).

The growth and maturation affect the cardiorespiratory response to exercise. In our experimental design, the adolescent groups were age matched without a maturity assessment as suggested by previous investigators (Mirwald et al. 2002; Barker et al. 2010b). Gender differences in biological maturation can reach its largest magnitude during adolescence with girls reaching mature height 2 years earlier than boys (Malina et al. 2004). A previous study in children and adolescent (8–18 year) evaluated oxygen delivery and efficiency by $\dot{V}O_{2}$/WR slope and stroke volume by $\dot{V}O_{2}$/HR slope in both males and females (Cooper et al. 2014). The maximal aerobic capacity and $\dot{V}O_{2}$/HR slope increased with age regardless of the gender and were both higher in boys than girls for all age groups investigated. Under the assumption that females would be more mature than male participants, the gender differences observed in our age‐matched study appear to be conservative. The gender differences between boys and girls with similar maturation level are expected to be higher than those observed in our study.

Another potential factor affecting the cardiovascular and respiratory responses of the girls of this study is the hormonal changes during the menstrual cycle which could have contributed to the variability in the physiological responses (Wheatley et al. 2014). Although, this study did not control for the phase of the menstrual cycle, a $\dot{V}O_{2}$ kinetics study in young women reported that high progesterone and estrogen level during the follicular and luteal phases of the menstrual cycle were not associated with changes in the regulation of $\dot{V}O_{2}$ kinetics and local muscle oxygen delivery at the onset of moderate exercise (Gurd et al. 2007).

Beside the empirical models used to characterize the kinetics of physiological variables and to identify the limitations to the physiological response, biophysical‐based mathematical models of the system can be used to describe the processes of oxygen transport and metabolism during exercise and analyze the effect HR kinetic parameters on $\dot{V}O_{2}$ kinetics. Previous studies applied mass balance relationships at steady state to quantify the extent of central and peripheral factors limiting maximal oxygen consumption at whole‐body level (Di Prampero and Ferretti 1990). To study the dynamics of oxygen uptake during muscle contraction mechanistic models that describe oxygen transport and metabolism in skeletal muscle at the cellular, tissue, and whole‐body levels can be used (Korzeniewski and Zoladz 2004; Lai et al. 2008a; Zhou et al. 2009; Rossiter 2011).

In summary, the dynamic responses to square‐wave exercise of moderate and heavy intensity in a group of female adolescents differ from those previously observed in male adolescents. Female adolescents consistently displayed slower $\dot{V}O_{2}$ kinetics at both exercise intensity. Although the primary gain is independent of exercise intensity, female adolescent showed a higher primary gain than male only at high exercise intensity providing evidences in gender differences in the energy cost. The faster HR kinetics in male than female and the association between HR and $\dot{V}O_{2}$ kinetics in male can in part explain the gender difference observed in $\dot{V}O_{2}$ kinetics. The HR changes relative to work rate are higher in female than male indicating gender differences in the mechanisms regulating the cardiorespiratory response to exercise.
